# The interplay between epidermal barrier distribution, microbiota composition, and immune infiltrate defines and stratifies psoriasis patients and is associated with disease severity

**DOI:** 10.1016/j.jtauto.2024.100257

**Published:** 2024-11-01

**Authors:** Elizabeth M. Ortega Rocha, Paul Hernández-Herrera, Sofia V. de los Santos- Carmona, Saraí G De León-Rodríguez, Ángel Juárez-Flores, Vadim Pérez-Koldenkova, Octavio Castro-Escamilla, Samira Muñoz-Cruz, Alicia Lemini-López, Laura C. Bonifaz

**Affiliations:** a Posgrado en Ciencias Biomédicas, Facultad de Medicina, Universidad Nacional Autónoma de México, Ciudad de México, Mexico; b Unidad de Investigación Médica en Inmunoquímica, UMAE Hospital de Especialidades, Centro Médico Nacional Siglo XXI, Instituto Mexicano del Seguro Social, Ciudad de México, Mexico; c Facultad de Ciencias, Universidad Autónoma de San Luis Potosí, San Luis Potosí, San Luis Potosí, Mexico; d Posgrado en Bioquímica, Facultad de Química, Universidad Nacional Autónoma de México, Ciudad de México, Mexico; e Posgrado en Ciencias Biológicas, Facultad de Medicina, Universidad Nacional Autónoma de México, Ciudad de México, Mexico; f Unidad de Investigación en Virología y Cáncer, Hospital Infantil de México Federico Gómez, Ciudad de México, Mexico; g Laboratorio Nacional de Microscopía Avanzada, Centro Médico Nacional Siglo XXI, Instituto Mexicano del Seguro Social, Ciudad de México, Mexico; h División de Investigación Clínica, Coordinación de Investigación en Salud, Instituto Mexicano del Seguro Social, Ciudad de México, Mexico; i Servicio de Dermatología, Hospital de Especialidades, Centro Médico Nacional Siglo XXI, Instituto Mexicano del Seguro Social, Ciudad de México, Mexico; j Coordinación de Investigación en Salud, Dirección de Prestaciones Médicas, Instituto Mexicano del Seguro Social, Ciudad de México, Mexico

**Keywords:** Psoriasis, Epidermal barrier, Skin microbiota, Psoriasis severity

## Abstract

Psoriasis is a chronic inflammatory autoimmune skin disease characterized by keratinocyte hyperproliferation, primarily driven by the IL-23/IL-17 axis. In addition to immune response, various skin components, including the epidermal barrier and the skin microbiota, have been individually implicated in the disease pathogenesis. Here, we aimed to investigate the interplay between epidermal tight junctions, Staphylococcus aureus enterotoxin B (SEB), and CD4 T cell-mediated immune responses. By immunofluorescence analyses of skin biopsies, we observed that claudin-1 distribution was significantly altered in psoriatic patients, which correlated with the localization of *Staphylococcus aureus* and SEB across skin layers and with disease severity. Furthermore, functional CD4 TCRvβ17 cells were associated with SEB presence in patients skin and positively correlated with psoriasis severity. Notably, in patients with SEB detected in the dermis, CD4 TCRvβ17 IL-17 cells were linked to barrier abnormalities.

Unsupervised analysis stratified psoriasis patients into three groups based on SEB presence and location, supporting the previous findings. The patient group with SEB in the dermis exhibited improved responses to biological therapy, including reductions in PASI score, claudin-1 fragmentation, *S. aureus* and SEB presence, and CD4 TCRvβ17 cell percentages. Our findings emphasize the complex interplay between epidermal barrier distribution, SEB localization, and functional CD4 TCRvβ17 cells in psoriatic skin, highlighting their potential in patient stratification in association with the severity of the disease.

## 1 Introduction

Psoriasis is a chronic inflammatory autoimmune skin disease, characterized by the hyperproliferation of keratinocytes, which promotes the formation of red plaques with silvery-white scales mainly on the skin of the elbows, lower back, and scalp [1]. The global prevalence is estimated at 2–3%, with over 60 million adults and children affected worldwide [2,3].

The pathogenic model of psoriasis involves the activation of the IL-23/IL-17 axis, which drives the pathogenic response of keratinocytes. Activation of plasmacytoid dendritic cells and myeloid dendritic cells by keratinocyte-derived antimicrobial peptides, and nucleic acids lead to the activation of autoreactive CD4 T cells. Additionally, IL-23 and IL-12 released by myeloid dendritic cells promote the differentiation of Th17 and Th1 cells, respectively, resulting in the secretion of IL-17, IFN-γ, and TNF-α. These cytokines act on keratinocytes stimulating the production and release of antimicrobial peptides, cytokines, and chemokines, amplifying the inflammatory response [4]. Furthermore, IL-17 signaling activates genes involved in keratinocyte proliferation and differentiation causing significant histological and functional alterations in the skin, including hyperkeratosis (thickening of the stratum corneum), acanthosis (thickening of epidermal layers), and parakeratosis (presence of nucleated corneocytes) [5,6]. Additionally, proteins critical in maintaining the skin barrier function are dysregulated [7].

The skin epidermal barrier comprises various structural, adhesive, and junctional proteins. Tight junction proteins, such as claudins and occludin, regulate the selective permeability of small molecules and water through the paracellular path within the epidermis [8]. The expression levels of tight junction proteins determine the skin epidermal barrier properties. For example, claudin-1 decreased expression in atopic dermatitis correlates with a dose-dependent increase in permeability to Biotin-556 tracer [9]. In psoriasis, the claudin-1 expression is decreased compared to healthy skin [10,11], although the precise mechanisms remain unclear. It is hypothesized that inflammatory cytokines, such as IL-17 and IFN-γ, may affect claudin-1 expression, as studies using skin models exposed to these cytokines showed decreased expression of this protein [12,13]. The altered expression of claudin-1 in psoriatic skin may disrupt the skin barrier function allowing antigens and microbial molecules to pass through the epidermis into the dermis, thereby contributing to the inflammatory response [14].

Other significant alterations observed in psoriatic skin are related to changes in the skin microbiota. The skin microbiota comprises a diverse community of bacteria, fungi, and viruses that contribute to maintaining tissue homeostasis by metabolizing natural skin compounds [15], resisting pathogen colonization [16], and interacting with keratinocytes and immune cells [17]. In healthy skin, the predominant bacteria phyla are *Actinobacteria* (51.8 %), *Firmicutes* (24.4 %), *Proteobacteria* (16.5 %), and *Bacteroidetes* (6.3 %). The three most prevalent genera are *Corynebacterium* (22.8 %; *Actinobacteria*), *Propionibacterium* (23.0 %; *Actinobacteria*), and *Staphylococcus* (16.8 %; *Firmicutes*). In contrast, the skin microbiota of psoriasis patients is less diverse than that of healthy individuals [18]. Notably, in psoriatic skin, there is a decrease in species from the phylum *Actinobacteria*, including those from the genus *Propionibacterium*, and an increase in species from the phylum *Firmicutes*, such as those from the genus *Staphylococcus* [19]. Within this genus, *Staphylococcus aureus* has been linked to psoriasis severity [20,21]. One study identified different strains of *S. aureus* in bacteria samples from psoriatic skin. These strains produced several exotoxins, including *S. aureus* enterotoxin A (SEA), *S. aureus* enterotoxin B (SEB), and *S. aureus* enterotoxin C (SEC). SEA and SEB were the most frequent exotoxins found in these cultures and the presence of *S. aureus* strains producing at least one exotoxin was correlated with the severity of the disease, as measured by the PASI [22]. In a previous study, we examined the SEB impact on the phenotype and function of CD4 lymphocytes from psoriasis patients. Our findings showed that stimulation with SEB led to an increase in the percentage of Th17 lymphocytes with a pathogenic phenotype (CD4 RORγt^+^ T-bet^+^), while the percentage of conventional Th17 lymphocytes (CD4 RORγt^+^ T-bet^-^) decreased. Furthermore, patients with extensive plaque lesions exhibited a higher percentage of pathogenic Th17 lymphocytes compared to patients with smaller plaque lesions [23]. This evidence strongly suggests that the presence of *S. aureus* and its exotoxins in the skin of patients with psoriasis could contribute to the disease pathogenesis by promoting the production of IL-17 and IFN-γ by Th17 lymphocytes. However, there was no direct evidence confirming the presence of *S. aureus* and its associated exotoxins in the patients dermis. Furthermore, the potential role of the abnormal expression of tight junction proteins, such as claudin-1, in mediating interactions between the skin microbiota and the immune system was not investigated.

One of the most recent hypotheses to explain the pathogenesis of non-communicable chronic inflammatory diseases is the “epithelial barrier hypothesis” [24], which explains that exposure to different kinds of allergens, pathogens, and environmental toxins causes epithelial barrier damage promoting the translocation of microbiota organisms and microbial components that leads to an inflammatory immune response. Several reports link epithelial barrier disruption and microbial dysbiosis to the altered immune response and pathogenesis of different diseases such as inflammatory bowel disease, asthma, and chronic rhinosinusitis [[25], [26], [27]]; however, less is known about how these factors impact on skin inflammatory diseases. The main studies on the skin epidermal barrier and skin microbiota in psoriasis patients describe the changes observed between disease and healthy conditions [28,29]. To fully understand the pathogenesis of psoriasis, it is important to investigate the potential link between the different components of the skin and the immune system. This will help to unravel the mechanisms driving the pathogenesis of the disease. This study aimed to assess the interplay between the epidermal barrier tight junctions, the microbiota, and the immune response mediated by CD4 T cells, in psoriasis pathogenesis.

## 2 Materials and methods

### 2.1 Psoriasis patients and healthy subjects

This study has been approved by the Ethics committee of the Hospital de Especialidades Centro Médico Nacional Siglo XXI and Centro Dermatológico Ladislao de la Pascua (registration number R-2017-785-058). We recruited 44 patients with plaque psoriasis diagnosis who had not received topic (within the previous 15 days) or systemic (within the previous two months) treatment. Supplementary Table 1 shows the demographic data of our group of patients. The recruitment process was conducted in accordance with the Helsinki statement. Volunteer patients signed the written informed consent after the protocol aim and procedure were explained to them. The treating physician evaluated the severity of the lesions by calculating the Psoriasis Area and Severity Index (PASI) and obtained the skin biopsy. Healthy control skin was obtained from patients who underwent gastrointestinal surgeries non-related to autoimmune disorders, cancer, or dermatological affections. Gastrointestinal surgery patients signed a written informed consent.

### 2.2 Skin sample collection and preparation

Lesional biopsies (6 mm) from patients and healthy skin were fixed in 4 % paraformaldehyde for later histological analysis. Fixed biopsies were dehydrated through serial solutions of 70 % EtOH, 96 % EtOH, dehydrated EtOH, and Xylene. Dehydrated tissues were embedded in paraffin and blocks were obtained and stored. Finally, 3 μm skin sections were obtained and placed in charged glass slides (Superfrost Plus Yellow) for further experiments.

### 2.3 Immunofluorescence assays and confocal microscopy

Paraffin was removed from the tissue by placing the slides into a stove (70 °C) for 40 min. Tissues were rehydrated in serial solutions of Xylene, 99 % EtOH, 80 % EtOH, 50 % EtOH, and distilled water. Heat-induced antigen retrieval was performed using a citrate buffer pH 6.0 (sodium citrate 10 μM) at 90 °C for 20 min. Then, tissue was permeabilized for 2h with a solution containing 10 mg/mL bovine serum albumin, 5 % horse serum, 0.02 % sodium azide, and 0.5 % Triton. According to the staining, permeabilized tissue was incubated overnight with primary antibodies, anti-claudin-1 (Abcam), anti-occludin (Abcam), anti-*Staphylococcus aureus* (Abcam), anti-enterotoxin B SEB (Abcam), anti-CD4 (Sigma Aldrich), anti-IL17 (RD Systems), anti-IFN-γ (BioLegend), or biotinylated anti-TCRvβ17 (Miltenyi). Incubation with secondary antibodies F(ab) anti-rabbit IgG-Alexa Fluor 488 (Jackson ImmunoResearch), anti-goat IgG-Alexa Fluor 594 (Jackson ImmunoResearch), or anti-mouse IgG-Alexa Fluor 647 (Jackson ImmunoResearch) was performed for 2h. When necessary, sections were incubated with Streptavidin-Alexa Fluor 594 (Jackson) for 2h. Nuclei were counterstained with Hoechst for 10 min (Invitrogen). Sections were finally mounted with Vectashield (Vector Laboratories) and stored at 4 °C until confocal analysis.

Micrographs were obtained on a Nikon Ti Eclipse inverted confocal microscope (Nikon Corporation) using NIS Elements v.4.50. Imaging was performed using a 20x (dry, NA 0.75) objective lens. Zoom was performed either at 3.4x, 4x, or with digital zoom. Images were analyzed using FIJI ImageJ Software (National Institutes of Health).

### 2.4 Tight junction image analysis

Fluorescence intensity was calculated on 20X images and normalized with Log10. To evaluate focal points on claudin-1 fluorescent staining, the average intensity of ten regions of interest (ROIs) corresponding to points located along the fluorescence signal, was calculated on 4x zoom images. Then, the ImageJ “Find Maxima” tool was used to localize and count the number of fluorescent points with an intensity value above the average. We will refer to this parameter as # maximum intensity points. Images that did not have enough fluorescence intensity to select the ten ROIs were excluded, whereby the analysis was performed in 33 of the 44 patients.

To evaluate the discontinuities, we used the following approach on claudin-1 and claudin-4 staining 20x images.

#### 2.4.1 Feature Extraction

The images were processed to extract numeric information for each channel. Each relevant structure was segmented, and features such as area, mean intensity, and average distance to the boundary were calculated for each segmented object. Additionally, the number of objects segmented in the image and the mean image intensity were also calculated.

#### 2.4.2 Object segmentation

The objects were segmented using a four-step approach: (1) Image boundary enhancement, (2) Detection of pixels in boundary and background, (3) Calculation of threshold for segmentation, and (4) Assignment of a label to each individual region.

1) Image Boundary Enhancement

The Laplacian filter, a second derivative filter, detects abrupt changes in intensity as zero-crossings, which is especially useful for detecting object edges. We employed the approach proposed by Hernandez-Herrera et al. [30], using a Laplacian at multiple scales to enhance edges for structures of different sizes.

2) Detection of Pixels in Boundary

According to the analysis by Hernandez-Herrera et al. [30], (1) bright objects have pixels with positive Laplacians, and (2) background pixels have oscillations between positive and negative values. Based on these properties, we selected background pixels as those with negative values.

3) Calculation of Threshold for Segmentation

From the detected background pixels, we obtained their intensity values from the original image. The threshold value for segmentation was determined as T = μ + 2σ, where μ is the mean and σ is the standard deviation of the intensity values detected in the previous step. We experimented with different thresholds, finding that T = μ + σ resulted in additional unwanted objects, while T = μ + 3σ missed some objects that should be included. Therefore, we fixed the threshold at T = μ + 2σ. Using this threshold, we created a binary image where pixels with a value of 1 correspond to the structures of interest (intensity value larger than T), and pixels with a value of 0 correspond to the background (intensity value lower or equal than T).

4) Assignment of a Label to Each Individual Region

We assigned a label to each object, ensuring that objects not connected to another object in a 3x3 window were considered separate. Classical algorithms for identifying connected components in a binary image [[31], [32], [33]] were used to assign a unique label to each object.

#### 2.4.3 Features calculated for each image

Measurements for each image were calculated based on the statistics of each detected object:

Mean Intensity: The mean intensity value of all the pixels in the image.

Number of Objects: The total number of connected components in the labeled image.

Mean Area: The average number of pixels assigned to each connected component.

Mean Intensity of Objects: By overlaying the objects on the original image, we calculated and averaged the intensity of the pixels belonging to each object.

Mean Distance: The distance from each pixel in the segmentation to the boundary was computed. We selected the top 50 % highest distances for analysis and calculated the mean value. From now on we will refer to this parameter as fluorescence width.

### 2.5 Quantitative analysis of fluorescent cells

Cell quantification was conducted using a previously described in-house machine learning method [34]. We employed our deep-learning neural network trained with the Cellpose architecture. The original machine-learning model was trained and validated on melanoma skin images and we adapted the flow threshold and cell probability threshold scores to identify nuclei in psoriatic skin. Nucleus masks were generated and used for marker classification with the Annotater plug-in of FIJI ImageJ Software (National Institutes of Health). We selected a dataset to train and validate each machine learning classifier for detecting positive and negative cells for a specific marker. Subsequently, we utilized the nucleus masks and the ML-classifiers to perform an automated batch analysis. To quantify the percentage of positive cells, the total number of cells positive for a specific phenotype was divided by the total cell count in the field and multiplied by 100. Three distinct fields were quantified per patient, and the median value is represented in the plots.

### 2.6 PCA-based clustering

Principal component analysis (PCA) and 3D PCA were performed using 12 variables measured in patients from clinical data and immunofluorescence staining. These variables were: claudin-1 number of objects, claudin-1 fluorescence intensity, claudin-1 fluorescence width, claudin-1 number of maximum intensity points, *S. aureus* presence, SEB presence, SEB location, %CD4 cells, %CD4 TCRvbeta17 cells, %CD4 TCRvbeta17 IL-17 cells, %CD4 TCRvbeta17 IFN-γ cells, and PASI. Variables related to *S. aureus* and SEB are categorical variables, so to construct the PCA, we assigned a numerical value to each category. Data processing and normalization were conducted by using R V.4.4.1. Visualization was generated using the factoextra library.

### 2.7 Statistical analysis

All data are presented as the median and IQR (interquartile range). Fluorescence comparisons were made between percentages of cells in healthy controls and lesional skin, or between SEB^−^, SEB^+^ epidermis, and SEB^+^ dermis groups of patients. Statistical analyses were performed by applying the Mann-Whitney *U* test for non-parametric data. The correlations between variables were obtained with the non-parametric Spearman test. All statistical analyses were performed using Prisma 8 Software (Graphpad).

## 3 Results

### 3.1 The aberrant distribution of claudin-1 in psoriatic skin is associated with disease severity

**Fig. 1:**
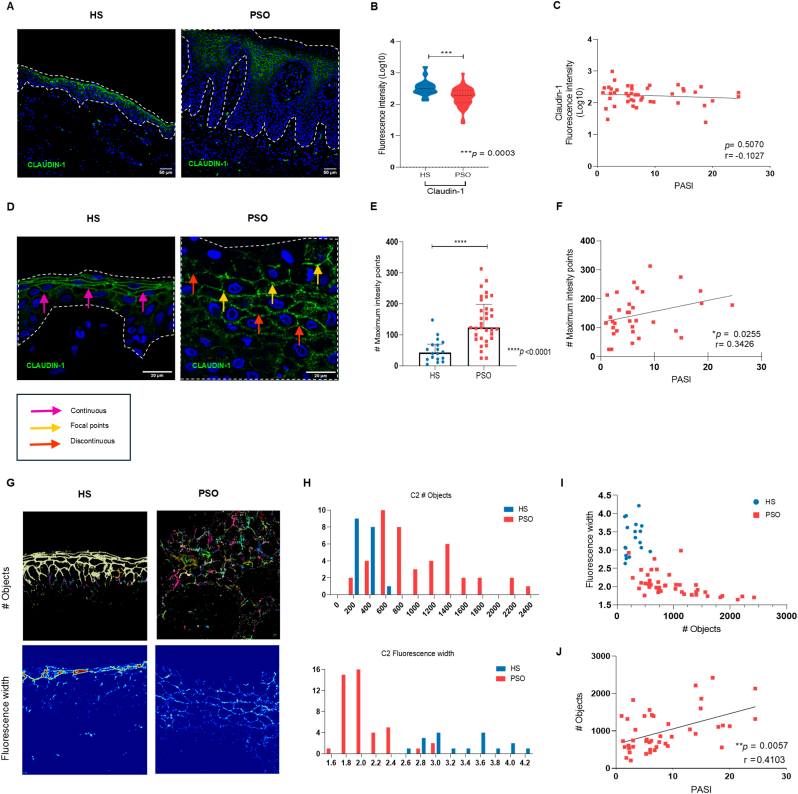
The distribution of claudin-1 in psoriasis skin is associated to disease severity. A) Immunofluorescence of claudin-1 in healthy and psoriatic skin. B) Fluorescence intensity of claudin-1, and C) claudin-1 correlation with the PASI score. D) Representative immunofluorescences of claudin-1 aberrant distribution in the skin from healthy subjects and psoriasis patients. E) Claudin-1 number of maximum intensity points in healthy and psoriatic skin and F) its correlation with the PASI score (n = 33). G) Representation of claudin-1 image segmentation analysis using the number of objects and the fluorescence width parameters in healthy and psoriatic skin. H) Frequency distribution of the two parameters (# of objects and fluorescence width) in healthy subjects and psoriasis patients. I) Scatterplot of fluorescence width vs # of objects in healthy subjects and psoriasis patients. J) Correlation of the # of objects with PASI score. HS: n = 18. PSO: n = 44, except for Fig. 1E and F.

To evaluate the state of the epidermal barrier in psoriatic skin, we performed an immunofluorescence assay to detect claudin-1, claudin-4, and occludin expression and compared it to healthy skin. In healthy skin, claudin-1 expression was located in the apical region, whereas in psoriatic skin it was widely expressed throughout the epidermis (Fig. 1A). Then, we measured the fluorescence intensity and found that claudin-1 expression was markedly reduced in psoriatic skin compared to healthy skin (Fig. 1B). As we found these differences, we asked whether claudin-1 fluorescence intensity could be associated with the psoriasis severity, but we found no correlation between this protein and the PASI (Fig. 1C). Claudin-4 expression was located on the apical region of the epidermis in healthy skin, and this localization was similar in psoriatic skin (Fig. S1A). There was no difference in the claudin-4 fluorescence intensity between healthy and psoriatic skin (Fig. S1B), and neither correlation with the PASI (Fig. S1C). Furthermore, occludin expression was located in the region corresponding to the stratum corneum in healthy skin, whereas in psoriatic skin, occludin had a wide expression throughout the epidermis (Fig. S1F). Occludin fluorescence intensity was higher in psoriatic skin compared with healthy skin (Fig. S1G), but it did not correlate with the PASI (Fig. S1H). Even though the levels of expression of these tight junction proteins in the epidermis were not associated with the disease severity, we observed relevant differences in the distribution of these proteins among the different PASI scores. Fig. 1D shows that in psoriatic skin claudin-1 was forming focal points (yellow arrows) of expression distributed along the keratinocyte membrane, although it can also be discontinuously distributed along the same membrane (red arrows). This is not the case for claudin-1 expression on healthy skin where the fluorescence intensity was continuous along the cell membrane (pink arrows).

To quantify claudin-1 expression and its distribution, we followed two distinct methodologies. First, we quantified the number of focal points defined as points with fluorescence intensity values above average. We found that psoriatic skin had a higher # of maximum intensity points compared with healthy skin (Fig. 1E), and this was slightly correlated with the PASI score (Fig. 1F). The second, was the quantification of the fluorescence discontinuities. Since the claudin-1 differences in distribution were challenging to analyze using conventional fluorescence image analysis methods, we chose to employ a computational and mathematical approach. We applied a Laplacian algorithm to segment and identify objects connected along claudin-1 fluorescence images and to measure fluorescence width. The number of objects indicates the degree of fragmentation or uniformity of fluorescence intensity across the epidermis, whereas the fluorescence width represents the distance between the pixels and boundary of the segmented objects. Fig. 1G shows representative image results obtained after applying the Laplacian algorithm. As this figure shows, healthy skin has few segmented objects throughout the epidermis (one color predominates in the image) whereas psoriatic skin has many segmented objects throughout the epidermis (various colors predominate in the image). Also, healthy skin has a wider fluorescence intensity than psoriatic skin. Fig. 1H shows the frequency distribution of the values obtained for these parameters in healthy subjects and patients. As shown in Fig. 1I, these two parameters separate and distinguish psoriasis patients from healthy subjects. We also evaluated whether claudin-1 aberrant distribution was associated with the disease severity, and we found a positive correlation between claudin-1 number of objects and the PASI score (Fig. 1J). Claudin-1 distribution was fragmented in psoriatic skin (measured by the number of objects) and was associated with disease severity. We also analyzed claudin-4 expression distribution using the same method; nonetheless, these two parameters could not separate psoriasis patients from healthy subjects (Fig. S1D), and they neither correlated with the PASI (Fig. S1E). We were unable to devise a methodology to analyze the occludin distribution due to its high variability in expression between patients. We found three different occluding distribution patterns in the skin of psoriasis patients; pattern 1 was characterized by an interrupted expression of occludin in the uppermost layer of the skin, pattern 2 was characterized by the expression of occludin in the cytosol of keratinocytes throughout the epidermis, and pattern 3 was characterized by no expression of occludin protein in the skin. Fig. S1I shows the three different occludin distribution patterns found in psoriatic skin. Altogether, these results indicate that the aberrant distribution of claudin-1, rather than merely its expression, is a hallmark of psoriatic skin associated with the disease severity.

### 3.2 The aberrant distribution of claudin-1 is related to *S. aureus* and SEB location on psoriatic skin

**Fig. 2:**
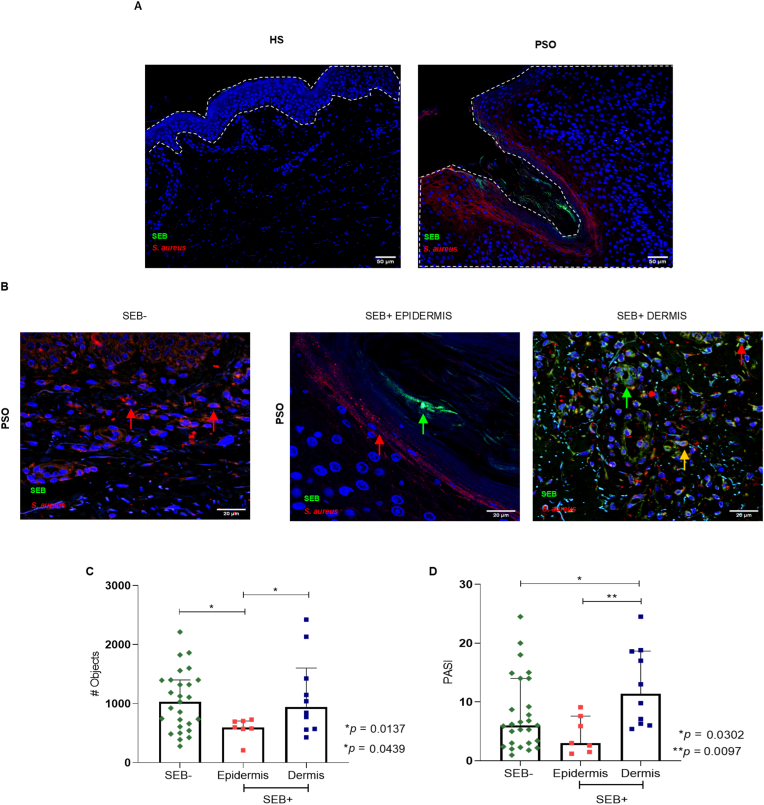
Claudin-1 number of objects is a parameter related to SEB location in psoriatic skin. Immunofluorescences of A) *S. aureus* and SEB in healthy (HS) and psoriasis (PSO) skin and B) *S. aureus* and SEB in the skin of SEB^−^ and SEB ^+^ psoriasis patients according to their location in the epidermis or dermis. The red arrows indicate *S. aureus* fluorescence signal, the green arrows indicate the SEB fluorescence signal, and the yellow arrow indicates the colocalization of both signals. C) Number of objects in the epidermis and dermis of SEB^−^, SEB^+^ epidermis, and SEB^+^ dermis patients and D) their corresponding PASI (n = 44).

Furthermore, we investigated whether the claudin-1 aberrant distribution could be related to the translocation of bacterial components towards the epidermis and dermis. To determine this, we evaluated the presence of *S. aureus* and its enterotoxin B (SEB) on psoriatic skin. This bacterium was present in almost all patients (except for one patient) and SEB was present in 17 of 44 patients, which represents 38.6 % of the total (we will refer to this group as SEB^+^ patients and as SEB^−^ patients for the rest of the patients) (Fig. 2A). We could distinguish three groups of patients according to *S. aureus* and SEB location: twenty-seven SEB^−^ patients with *S. aureus* localized in the dermis, seven patients with both *S. aureus* and SEB localized in the epidermis, and ten patients with both *S. aureus* and SEB localized in the dermis (we will refer to these SEB^+^ subgroups as SEB^+^ epidermis and SEB + dermis patients, respectively) (Fig. 2B).

SEB is a protein with a molecular size of 28 kDa [35]. Due to its size, the translocation of SEB through the skin could be regulated by the barrier formed by tight junctions. Next, we evaluated if the location of *S. aureus* and SEB in the epidermis or dermis was related to claudin-1 aberrant distribution by comparing the number of objects between the three groups of patients. Fig. 2C shows that SEB^−^ and SEB^+^ dermis patients had similar number of objects. Interestingly, SEB^+^ epidermis patients had fewer objects compared with the other groups. Importantly, patients with SEB located on the dermis had higher PASI scores than the other groups (Fig. 2D). We also compared claudin-1 fluorescence intensity between SEB^−^ and SEB ^+^ subgroups of patients to evaluate whether it was related to *S. aureus* and SEB location on the skin. We did not find any difference in claudin-1 fluorescence intensity between the three groups of patients (Fig. S2A), which supports our previous result that claudin-1 distribution, rather than just protein expression, is related to the disease pathogenesis.

These results show that claudin-1 aberrant distribution was associated with the *S. aureus* and SEB location in psoriatic skin.

### 3.3 The presence of CD4 TCRvβ17 IL-17^+^ cells correlate with SEB presence and disease severity

**Fig. 3:**
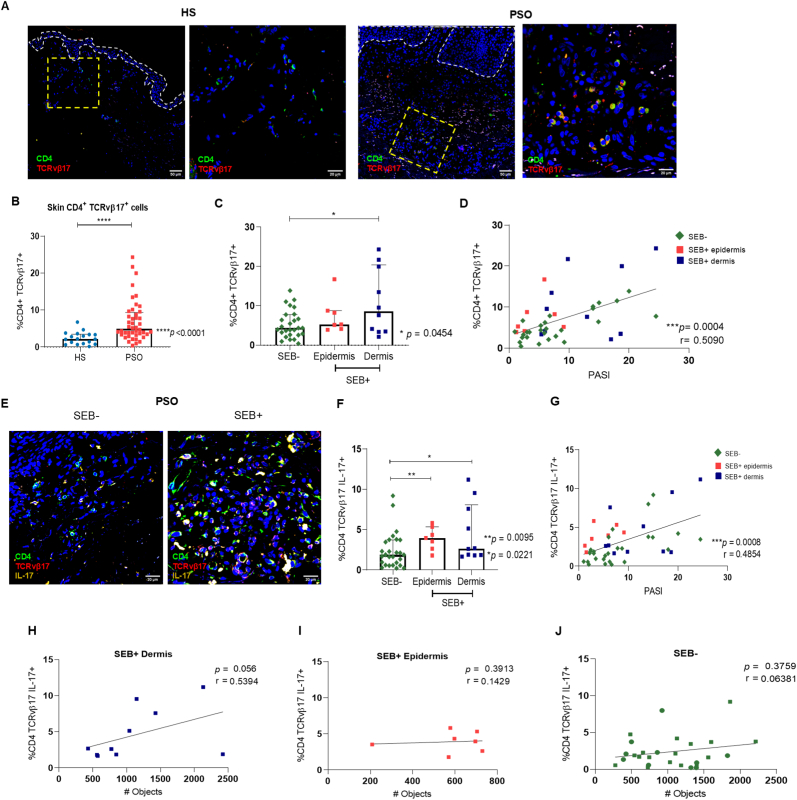
The percentage of CD4 TCRvβ17 IL-17 cells is positively correlated with the psoriasis severity. A) Immunofluorescences and B) percentages, of CD4 TCRvβ17 cells in healthy (n = 18) and psoriasis skin (n = 44). C) Frequency of CD4 TCRvβ17 cells in SEB^−^, SEB^+^ epidermis, and SEB^+^ dermis patients and D) their correlation with the PASI score (green for SEB^−^ patients, red for SEB^+^ epidermis patients and blue for SEB^+^ dermis patients, n = 44). E) Immunofluorescences of CD4 TCRvβ17 IL-17 cells of SEB^−^, SEB^+^ epidermis, and SEB^+^ dermis patients, F) its quantification, and G) correlation with the PASI score (n = 44). H) Correlation of CD4 TCRvβ17 IL-17 cells with the claudin-1 number of objects in SEB^+^ dermis patients (n = 10), in I) SEB^+^ epidermis patients (n = 7) and J) in SEB^−^ patients (n = 27).

To assess if the SEB presence in psoriatic skin was associated with the CD4 T cell inflammatory response. First, we evaluated the CD4 cell infiltrate and found that psoriatic skin had a higher percentage of CD4 cells compared with healthy skin (Figs. S3A and S3B). Next, we compared the percentage of CD4 cells between SEB^−^ and SEB^+^ subgroups of patients and found no difference between these groups (Fig. S3C); however, the percentage of CD4 cells had a slight correlation with the disease severity (Fig. S3D). Given that SEB is an enterotoxin that functions as a superantigen and expands different TCRvβ families [36], we evaluated the CD4 TCRvβ17 clone in patients skin. Fig. 3A shows the immunofluorescence of CD4 TCRvβ17 cells in healthy and psoriatic skin, where psoriatic skin had a higher percentage of CD4 TCRvβ17^+^ cells than healthy skin (Fig. 3B). When we compared SEB^−^ and SEB^+^ subgroups of patients, SEB^+^ dermis showed a higher percentage of this cell population than SEB^−^ patients (Fig. 3C). There were no differences in the TCRvβ17 cells between the groups with SEB localized in epidermis or dermis. Importantly, the percentage of CD4 TCRvβ17^+^ cells in psoriatic skin correlated with the PASI (Fig. 3D). Then, we evaluated the cytokine production of CD4 TCRvβ17 cells and found significant IL-17 and IFN-γ expression (Fig. 3E and S3E). SEB^+^ epidermis and SEB^+^ dermis patients had a higher percentage of CD4 TCRvβ17 IL-17^+^ cells than SEB^−^ patients (Fig. 3F), and the percentage of these cells in psoriatic skin also had a positive correlation with the PASI (Fig. 3G). On the other hand, there was no difference in the percentage of CD4 TCRvβ17 IFN-γ^+^ cells between SEB^−^ and SEB^+^ epidermis patients (Fig. S3F), but SEB^+^ dermis patients had a higher percentage of these cells than SEB^−^ patients. Furthermore, we asked if the CD4 cell infiltrate found in SEB^−^ and SEB^+^ patients was correlated with the claudin-1 distribution. According to the previously mentioned results and Fig. 2C, a slight positive correlation between CD4 TCRvβ17 IL-17^+^ and claudin-1 number of objects was only found in SEB^+^ dermis patients (Fig. 3H), but not in SEB^+^ epidermis patients (Fig. 3I) neither SEB^−^ patients (Fig. 3J). This was not the case for CD4 TCRvβ17 IFN-γ^+^ cells and claudin-1 number of objects in SEB^−^ and SEB^+^ subgroups of patients (Figs. S3G–I). These results suggest that the presence of SEB on psoriasis patients skin is associated with the increase of CD4 TCRvβ17 IL-17^+^ cells, and this percentage correlates positively with the disease severity.

### 3.4 Claudin-1 distribution, SEB and CD4 TCRvβ17 cells characterize and stratify psoriasis patients

**Fig. 4:**
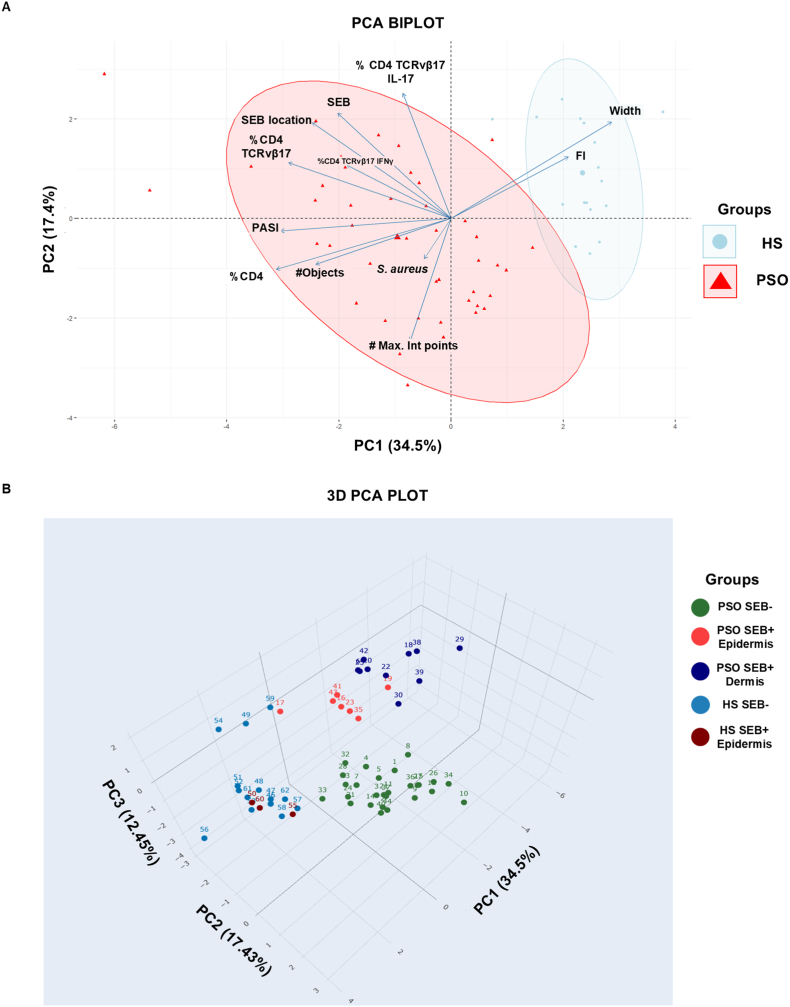
Claudin-1 distribution, SEB, and CD4 TCRvβ cells characterize psoriasis skin and segregate patients into three groups. A) Principal component analysis includes data from 44 psoriasis patients and 18 healthy subjects. B) 3D visualization of principal component analysis with patients anottation according to SEB presence and location. The 12 variables evaluated were: claudin-1 fluorescence width (Width), claudin-1 fluorescence intensity (FI), claudin-1 number of objects (#Objects), claudin-1 number of maximum intensity points (#Max. int. points), *S. aureus* presence, SEB presence, SEB location, percentage of CD4 cells, percentage of CD4 TCRvβ17 cells, percentage of CD4 TCRvβ17 IL-17 cells, percentage of CD4 TCRvβ17 IFN-γ cells, and PASI score. HS=Healthy skin, PSO= Psoriatic skin.

Then, we assessed which of the variables identified in this study were associated with psoriasis patients and which ones were with healthy subjects. To determine this, we did a principal component analysis (PCA) using the 12 variables analyzed in this study (Fig. 4A). The PCA plot shows the segregation of healthy subjects from patients (mainly separated by PC1 explaining 34.5 % of variability), where the claudin-1 fluorescence width and fluorescence intensity are the principal components that define healthy subjects. Whereas the principal components that define psoriasis patients along PC1 are the PASI score and the percentage of CD4 cells (see Supplementary Table 2 for variables weight values). For psoriasis patients, we found an interesting distribution along PC2, where some patients located above this axis and other patients located beneath it. The components that define patients from the upper right quadrant are the percentage of CD4 TCRvβ17 IL-17, the categorical variables SEB presence and location, and claudin-1 fluorescence width, while the components that define patients from the lower quadrants are the claudin-1 number of maximum intensity points, the percentage of CD4 cells and claudin-1 number of objects. To describe better the segregation observed between patients, we performed a 3D PCA and annotated each patient in the plot to identify which state of the categorical variables SEB presence and location (SEB negativity or SEB positivity; SEB in the epidermis or SEB in the dermis) was related with the observed results (Fig. 4B). The 3D PCA plot shows the segregation of two main groups of patients at the upper region and lower region of the plot. Interestingly, the patients grouped in the upper region of the 3D plot correspond to SEB^+^ patients, while the patients grouped in the lower region correspond to SEB^−^ patients. Within SEB^+^ patients, we found the segregation of SEB^+^ epidermis patients (in the lower region of SEB^+^ group) and SEB^+^ dermis patients (in the upper region of SEB^+^ group). This unsupervised PCA analysis supports our previous results where three groups of patients, SEB^−^, SEB^+^ epidermis, and SEB^+^ dermis patients, were identified. These results strongly suggest that the claudin-1 distribution, the presence and location of SEB, and the infiltrate of CD4 TCRvβ17 IL-17 cells are parameters that define and segregate psoriasis patients.

### 3.5 Biological therapy modifies the claudin-1 distribution and reduces the presence of *S. aureus* and CD4 TCRvβ17 infiltrate in psoriasis patients with SEB located in the dermis

**Fig. 5:**
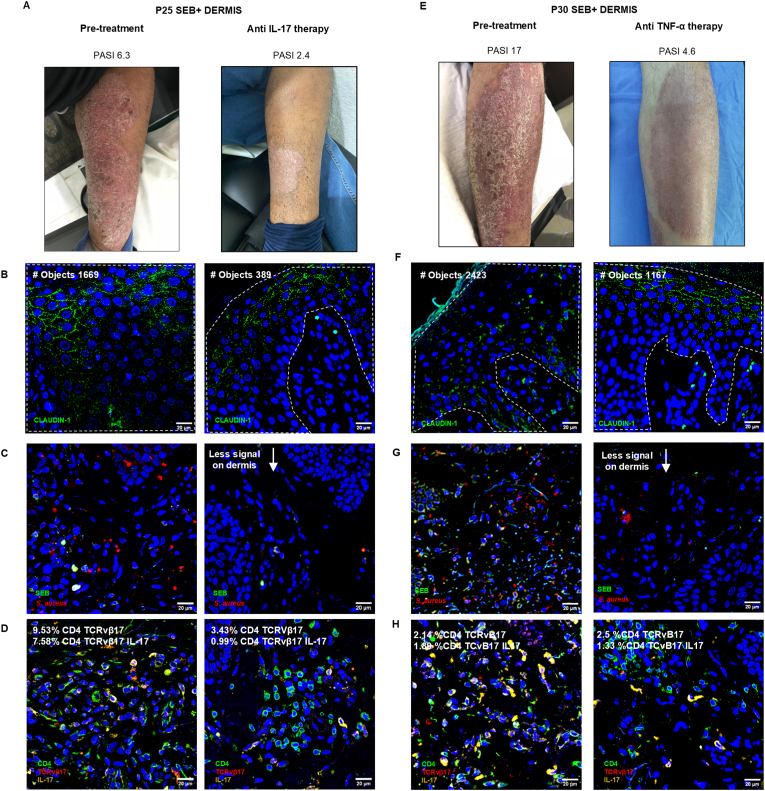
Biological therapy modifies claudin-1 distribution, S. aureus, SEB, and CD4 TCRvβ17 infiltrate in psoriatic skin. A) Representative photographs of skin lesion of SEB + dermis psoriasis patient pre-treatment (PASI 6.3) and after anti-IL-17 Secukinumab therapy (PASI 2.4). B) Immunofluorescence images of claudin-1 distribution with the number of objects value pre-treatment and after anti-IL-17 therapy. C) Immunofluorescence images of *S. aureus* and SEB pre-treatment and after anti-IL-17 therapy. D) Images of CD4 TCRvβ17 IL-17+ cells with percentage values of CD4 TCRvβ17 cells and CD4 TCRvβ17 IL-17 cells, pre-treatment and after anti-IL-17 therapy. E) Representative photographs of a skin lesion of SEB + dermis psoriasis patient pre-treatment (PASI 17) and after anti-TNF-α Infliximab therapy (PASI 4.6). F) Immunofluorescence images of claudin-1 distribution with the number of objects value pre-treatment and after anti-TNF-α therapy. G) Immunofluorescence images of *S. aureus* and SEB pre-treatment and after anti-TNF-α therapy. H) Images of CD4 TCRvβ17 IL-17+ cells with percentage values of CD4 TCRvβ17 cells and CD4 TCRvβ17 IL-17 cells, pre-treatment and after anti-TNF-α therapy.

Finally, to evaluate if the treatment with monoclonal antibodies, which improved the clinical psoriasis features measured by PASI score reduction, affects the different components evaluated (barrier, microbiota, and CD4 cell infiltrate), we evaluated these components in patients treated with the monoclonal antibody anti-IL17 (Secukinumab) or anti-TNF-α (Infliximab or Adalimumab). We present the case of a psoriasis patient with plaques in the arms and legs, with a PASI score of 6.3, and with the presence of SEB in the dermis. We evaluated the claudin-1 distribution, and the presence of *S. aureus*, SEB, and CD4 TCRvβ17 cells in the patient skin biopsy before and after Secukinumab therapy. Fig. 5A left shows a photograph of a lesion in the leg at baseline, where claudin-1 distribution was characterized by 1669 objects (Fig. 5B left), which represents the fragmentation of claudin-1 in the epidermal barrier. In addition, we found the presence of *S. aureus* and SEB in the dermis (Fig. 5C left). In the lesional skin, we found 9.53 % of CD4 TCRvβ17 cells and 7.58 % of CD4 TCRvβ17 IL-17 cells (Fig. 5D left). This patient received Secukinumab and his condition improved to PASI 0 within 12 weeks of treatment, and its administration continued for 34 months. After this period, the lesions reactivated and reached a PASI 2.4, and the treating physician took a biopsy for further analysis. Fig. 5A right shows a photograph of the same leg lesion at that time, where the claudin-1 number of objects decreased to 389 compared to the number of objects at baseline, which shows a less fragmented barrier in a lower PASI (Fig. 5B right). We also found less signal of *S. aureus* and SEB on the dermis of this patient (Fig. 5C right), and a decrease in the percentage of CD4 TCRvβ17, and CD4 TCRvβ17 IL-17 (decreased to 3.43 % and 0.99 %, respectively, compared with baseline) (Fig. 5D right).

We evaluated the case of another SEB^+^ dermis psoriasis patient who had a PASI score of 17, with the presence of plaques in the arms and legs. We evaluated the same parameters mentioned earlier, both before and after Infliximab therapy. Fig. 5E left shows a photograph of a lesion in the leg at baseline. In this patient, claudin-1 distribution was characterized by 2423 number of objects (Fig. 5F left). Furthermore, we found the presence of *S. aureus* and SEB in the dermis (Fig. 5G left). Accordingly, there were 2.14 % of CD4 TCRvβ17 cells and 1.89 % of CD4 TCRvβ17 IL-17 cells in the patient skin (Fig. 5H left). This patient received Infliximab, and his condition improved to PASI 0 within 30 weeks of therapy, with a dose adjustment at 18 weeks. The therapy administration continued, and after 32 months the lesions reached a PASI 4.6. The treating physician took a biopsy for further analysis. Fig. 5E right shows a photograph of the same leg lesion at that time. At PASI 4.6, the claudin-1 number of objects decreased to 1167 compared to the number of objects at baseline (Fig. 5F right). We found less signal for *S. aureus* and SEB on the dermis of this patient (Fig. 5G right), a slight increase in CD4 TCRvβ17 cells, and a slight decrease of CD4 TCRvβ17 IL-17 cells to 2.5 % and 1.33 %, respectively, compared to baseline (Fig. 5H right).

Lastly, we evaluated the case of a SEB^+^ epidermis psoriasis patient to contrast our previous results. This patient had a PASI score of 7.6 with the presence of plaques in the trunk, arms, and legs. We evaluated the same parameters aforementioned, both before and after Adalimumab therapy. Supplementary Fig. 4A left, shows a photograph of a lesion in the back at baseline. In this patient, claudin-1 distribution was characterized by 704 objects, which is a lower baseline value compared to SEB^+^ dermis patients (Fig. S4B left). Furthermore, we found the presence of *S. aureus* and SEB in the epidermis (Fig. S4C left). Accordingly, there were 8.23 % of CD4 TCRvβ17 cells and 5.33 % of CD4 TCRvβ17 IL-17 cells in the patient's skin (Fig. S4D left). This patient received anti-TNF-α therapy (Adalimumab), and his condition improved to PASI 5.2 within 4 weeks of therapy. The therapy administration continued with favorable outcome, and after 38 months the lesions reached a PASI 4.3. The treating physician took a biopsy for further analysis. Fig. 4A right shows a photograph of a lesion in the lower back at that time. At PASI 4.3, the claudin-1 number of objects was 889. The number of objects at this time was not affected compared to the number of objects at baseline, contrary to what we observed for SEB^+^ dermis patients (Fig. S4B right). Nevertheless, we found less signal for *S. aureus* and SEB on the epidermis of this patient (Fig. S4C right) and a decrease in CD4 TCRvβ17 cells and CD4 TCRvβ17 IL-17 cells to 5.01 % and 1.77 %, respectively, compared to baseline (Fig. S4D right). These results suggest that although this patient shows less affection in the barrier, the activity of the disease related to the presence of SEB in the epidermis and the expansion of TCRVβ17 clones can be restored after biological treatment. This also shows that SEB^+^ epidermis and SEB^+^ dermis patients belong to different groups that appear to respond differently to biological therapy. Altogether, these results suggest that the decrease in the PASI score by anti-IL-17 or anti- TNF-α therapy is associated with a recovery of the barrier distribution and a decrease in the microbiota components and immune infiltrate cells in SEB^+^ dermis patients.

## 4 Discussion

Psoriasis is a complex chronic inflammatory autoimmune disease involving various skin components. The prevailing pathogenesis model for psoriasis relies on the IL-17/IL-23 axis, which explains the major phenotypic and clinical alterations observed in the skin of affected individuals [4]. The skin is a complex organ, encompassing diverse components such as the epidermal barrier and the microbiota, both of which have the potential to contribute to the disease pathogenesis. Previous studies have focused on understanding the role of these individual components; however, to fully understand the pathogenesis of such a complex disease, it is necessary to evaluate the interactions and relationships between these key components.

Remarkably, our results indicate that psoriasis patients can be distinguished from healthy donors by evaluating tight junctions, microbiota, and immune infiltrate. Notably, these components segregate patients into three groups based on the degree of barrier disruption, the presence and localization of SEB, and the percentage of CD4 TCRvβ17 cells related to disease activity.

Previous studies had examined tight junction proteins expression to describe differences between healthy and psoriatic skin [10,29,37]. However, these studies did not evaluate the correlation between the protein expression levels and disease severity or other clinical parameters. Our data are consistent with previous reports, showing a reduced expression of claudin-1 in psoriatic skin compared to healthy skin. To assess the correlation between claudin-1 expression and the disease severity, we initially used fluorescence intensity as a conventional imaging parameter. Due to the complexity of the images, this approach proved inadequate for quantifying the changes observed in the fluorescence images. Therefore, we employed a mathematical and computational approach, specifically a Laplacian algorithm [30], to quantify the differences between images from psoriatic and healthy skin, and to correlate these differences with disease severity. This novel approach provides a more precise evaluation of tight junction expression and distribution in the skin, allowing further analysis and comparisons with other disease parameters. Our results indicate that claudin-1 is an important tight junction protein in psoriasis pathogenesis, as its aberrant distribution is associated with disease severity.

Claudin-1 downregulation in cultured keratinocytes has been associated with tight junction dysfunction mainly by increasing the permeability to ions such as Na^+^, Cl^−^, and Ca^2+^, as well as to molecular tracers with distinct molecular weights (ranging from 4 kDa to 40 kDa) [8]. Given that microbial compounds like SEB have a molecular weight of 28 kDa [38], the fragmentation of claudin-1 could lead to increased permeability and translocation of these compounds. Although claudin-4 and occludin also contribute to barrier function in the skin, their downregulation is associated with increased permeability to molecular traces of lower molecular weight (4 kDa) [8]. In a similar study performed on skin samples from healthy subjects and atopic dermatitis patients, a biotin-556 tracer was injected into skin biopsies and the paracellular permeability was measured by counting the regions where the tracer colocalized with claudin-1 fluorescence (“tracer stops”). The results showed a decrease in tracer stops in atopic dermatitis skin compared to healthy skin correlating with claudin-1 fluorescence intensity [9]. In our study, we investigated claudin-1 distribution in relation to barrier dysfunction by evaluating the translocation of microbiota components through the skin. We specifically focused on *S. aureus* and SEB due to previous reports suggesting their possible participation in psoriasis pathogenesis [19,23]. To our knowledge, this is the first study where the presence of *S. aureus* and SEB in the dermis of psoriasis patients is described. There are some studies where bacteria are isolated from psoriatic or atopic dermatitis skin by performing skin swabs [21,22]. This technique allows the culture of superficial bacteria, whereas an immunofluorescence assay allows the identification and location of bacteria in the tissue. By using immunofluorescence, we identified and located *S. aureus* and SEB in the epidermis and dermis of patients and distinguished three groups of psoriasis patients with different involvement of the barrier integrity given by claudin-1. The aberrant distribution of claudin-1 observed in the groups of patients could be influenced by different factors. For instance, it has been described that IL-1β downregulates claudin-1 expression *in-vitro* in HaCat cell cultures [10]. In another study, primary human keratinocyte cultures were stimulated with a pro-differentiation high Ca^2+^-containing medium, which increased the claudin-1 localization in the cell membrane [39]. Therefore, the process of differentiation in psoriasis skin and the signaling associated with it, could contribute to claudin-1 aberrant distribution. In this group of patients, we did not identify the factors associated with the claudin-1 aberrant distribution, but we found that it is associated with an abnormal location of both *S. aureus* and SEB. Furthermore, patients with SEB dermal location were associated with greater disease severity. We are aware that other bacterial species and microbial compounds may also contribute to the pathogenesis of psoriasis. Our study paves the way for further research into the roles of different skin bacterial species and their compounds in psoriasis. For instance, species from the genus *Corynebacterium* have been reported to increase in their relative abundance in psoriatic skin [40].

Following the identification of *S. aureus* and SEB in psoriatic skin, we further evaluated their impact on the immune response. Since SEB is a superantigen, we investigated the presence of TCRvβ17 clones that may have been expanded by SEB exposure. These clones were found to correlate with disease severity and exhibited functional characteristics, including the expression of IL-17. Our study is the first to establish a correlation between *S. aureus* and SEB with the immune infiltrate in psoriatic human skin. Previous works have related *Streptococcus* with the onset of guttate psoriasis [41]. It has been described that the development of skin lesions could be due to the activation of antigen-specific clones targeting bacterial molecules that resemble skin molecules, such as the M protein, which is similar to skin keratin. Nonetheless, our findings indicate that the mechanism described in this study seems to be directed towards the expansion of TCRvβ17 clones, regardless of antigenic specificity. However, further research is required to verify this assertion. The presence of CD4 TCRvβ17 cells and the expression of IL-17 might be an additional mechanism contributing to plaque psoriasis pathogenesis, rather than serving as the initiator of inflammation. Furthermore, the presence of TCRvβ17 cells in the skin of psoriasis patients was related to the presence of SEB and seemed to be independent of SEB location. An important limitation of our study is the number of patients in the SEB^+^ subgroups, which could explain why we did not find differences in the CD4 TCRvβ17 infiltrate according to the SEB location. Even though, we found a positive correlation between CD4 TCRvβ17 IL-17 clones and claudin-1 barrier integrity only in patients with SEB dermal location, suggesting that this group has the greatest affection.

The parameters outlined in this study are useful for patient stratification. Traditional clinical and histological evaluations remain the standard methods to identify and diagnose psoriasis [50]. Clinical assessments of patients and the PASI are typically used to stratify patients into mild or moderate to severe disease categories, thereby guiding treatment decisions. Nevertheless, we described parameters different from PASI that helped us to stratify patients into three groups, mainly by the SEB presence and location. Further research will be needed to validate these parameters as potential disease biomarkers, which could help design novel treatment strategies.

Therapy selection for psoriasis patients is based on the severity of the lesions and the percentage of affected body surface area. For mild disease, treatment options include topical corticosteroids and phototherapy, while treatment options for moderate to severe disease include oral medication and biologic therapy directed against IL-17, IL-23, or TNF-α [42]. The use of anti-IL17 monoclonal antibodies has shown great efficacy in psoriasis patients [[43], [44], [45]], besides the reduction in PASI scores, this therapy has been shown to influence various disease parameters, such as skin microbiota composition [46], histological features [47], and immune-related genes [47]. In our study, we observed a decrease in the claudin-1 number of objects, the presence of *S. aureus* and SEB, and the percentage of CD4 TCRvβ17 clones, all correlated with a reduction in PASI scores. This suggests that the epidermal barrier, skin microbiota, and immune infiltrate are dynamic components involved in psoriasis pathogenesis. Despite the efficacy of IL-17 biologics, approximately 20 % of patients do not respond to these therapies as expected or stop responding to therapy over time. Our findings open the possibility of exploring new therapeutic targets directed against epidermal barrier components or microbial factors to aid in patients treatment. The use of combined therapies has been proven to be effective in clinical trials. For instance, the percentage of patients that achieved a PASI 75 was higher after combination therapy with Etanercept and methotrexate compared to monotherapy [48]. Although combination therapies with anti-IL-17 biologics are less studied, there is one report including 4 patients with recalcitrant psoriasis who achieved a PASI 75 reduction after treatment for 6–9 months with Secukinumab and Apremilast (phosphodiesterase-4 inhibitor) [49]. To date, there are no clinical trials that combine immune system blockade with therapies aimed at restoring the epidermal barrier or modifying the composition of the skin microbiota. Incorporating these critical aspects of skin health into the development of new therapies could lead to better treatment outcomes and potentially extend remission periods for patients.

## 5 Conclusion

This study underlines the relevance of the interplay between epidermal barrier distribution, the presence of microbiota components, and the immune infiltrate in defining and segregating psoriasis patients. These factors are also associated with disease severity.

## Funding sources

This work was funded by Consejo Nacional de Humanidades, Ciencia y Tecnología, CONAHCYT FONSEC SSA/IMSS/ISSSTE (S00082017/1–290138). This work has been made possible in part by grant number 2023-329644 from the 10.13039/100014989Chan Zuckerberg Initiative DAF, an advised fund of Silicon Valler 10.13039/100008174Community Foundation.

## CRediT authorship contribution statement

**Elizabeth M. Ortega Rocha:** Writing – original draft, Visualization, Investigation, Formal analysis. **Paul Hernández-Herrera:** Software, Methodology, Formal analysis, Data curation. **Sofia V. de los Santos- Carmona:** Investigation. **Saraí G De León-Rodríguez:** Formal analysis. **Ángel Juárez-Flores:** Formal analysis. **Vadim Pérez-Koldenkova:** Methodology. **Octavio Castro-Escamilla:** Writing – review & editing, Supervision. **Samira Muñoz-Cruz:** Writing – review & editing, Supervision. **Alicia Lemini-López:** Supervision, Conceptualization. **Laura C. Bonifaz:** Writing – review & editing, Supervision, Conceptualization.

## Declaration of competing interest

The authors declare that they have no known competing financial interests or personal relationships that could have appeared to influence the work reported in this paper.

## Data Availability

The link to the code we used for tight junction analysis is provided.
