# Supplementary material for: The interplay between epidermal barrier distribution, microbiota composition, and immune infiltrate defines and stratifies psoriasis patients and is associated with disease severity
Source: J Transl Autoimmun. 2024 Nov 1;9:100257. doi: 10.1016/j.jtauto.2024.100257 (PMC11584942; doi:10.1016/j.jtauto.2024.100257)
Supplement: Multimedia component 1 [file mmc1.docx]

**Supplementary figures**

***Supplementary table 1. Demographic data of psoriasis patients***

| Patient | Sex | Age | PASI |
| --- | --- | --- | --- |
| 1 | Female | 46 | 15 |
| 2 | Female | 38 | 0.98 |
| 3 | Male | 63 | 5.3 |
| 4 | N.D. | N.D. | 20 |
| 5 | Female | 36 | 18 |
| 6 | Male | 78 | 13 |
| 7 | Female | 47 | 2.3 |
| 8 | Female | 41 | 6.8 |
| 9 | Female | N.D. | 14 |
| 10 | Female | 57 | 9.2 |
| 11 | Male | 58 | 5.9 |
| 12 | Male | 49 | 14.9 |
| 13 | Male | N.D. | 8.7 |
| 14 | N.D. | N.D. | 3.4 |
| 15 | Female | 23 | 1.8 |
| 16 | Male | 31 | 3 |
| 17 | Female | 56 | 2.6 |
| 18 | Male | 59 | 9.8 |
| 19 | Male | 37 | 5.9 |
| 20 | Male | 26 | 7.1 |
| 21 | Male | 21 | 3 |
| 22 | Male | 66 | 5.4 |
| 23 | Male | 82 | 1.5 |
| 24 | Female | 52 | 2.2 |
| 25 | Male | 36 | 6.3 |
| 26 | Female | 48 | 24.5 |
| 27 | Male | 45 | 7.8 |
| 28 | Male | 51 | 6 |
| 29 | Male | 56 | 18.8 |
| 30 | Male | 55 | 3.4 |
| 31 | Female | 57 | 2 |
| 32 | Male | 55 | 14 |
| 33 | Female | 42 | 2.4 |
| 34 | Male | 46 | 6.2 |
| 35 | Male | 72 | 1.2 |
| 36 | Male | 62 | 5.3 |
| 37 | Male | 37 | 3 |
| 38 | Female | 70 | 24.5 |
| 39 | Male | 63 | 18.6 |
| 40 | Female | 80 | 6.6 |
| 41 | Male | 69 | 7.6 |
| 42 | Male | 50 | 6 |
| 43 | Male | 39 | 9.1 |
| 44 | Male | 54 | 5 |

*N.D= not determined*

***Supplementary table 2. PCA weight values***

| **Variables** | **PC1** | **Variables** | **PC2** | **Variables** | **PC3** |
| --- | --- | --- | --- | --- | --- |
| Width | 0.36658747 | %CD4 TCRvβ17 IL-17 | 0.45250101 | # Objects | 0.37140936 |
| Claudin-1 FI | 0.26789169 | SEB | 0.38125887 | %CD4 TCRvβ17 IL-17 | 0.2781675 |
| *S. aureus* | -0.06169085 | Width | 0.34853542 | PASI | 0.25771173 |
| # Max. Int. Points | -0.09343817 | SEB location | 0.34671077 | %CD4 TCRvβ17 | 0.18808526 |
| %CD4 TCRvβ17 IL-17 | -0.10981037 | Claudin-1 FI | 0.22261799 | %CD4 TCRvβ17 IFNγ | 0.10748116 |
| %CD4 TCRvβ17 IFNγ | -0.23862429 | %CD4 TCRvβ17 | 0.20204107 | Claudin-1 FI | 0.06277078 |
| SEB | -0.2590703 | %CD4 TCRvβ17 IFNγ | 0.19398716 | Width | 0.03053426 |
| # Objects | -0.30874285 | PASI | -0.04503566 | % CD4 | -0.04196682 |
| SEB location | -0.3175986 | *S. aureus* | -0.14549499 | # Max. Int. Points | -0.21929078 |
| %CD4 TCRvβ17 | -0.37137801 | # Objects | -0.16574048 | SEB location | -0.39515856 |
| PASI | -0.38962087 | % CD4 | -0.18409897 | SEB | -0.46211185 |
| % CD4 | -0.39909505 | # Max. Int. Points | -0.44230024 | *S. aureus* | -0.49690494 |


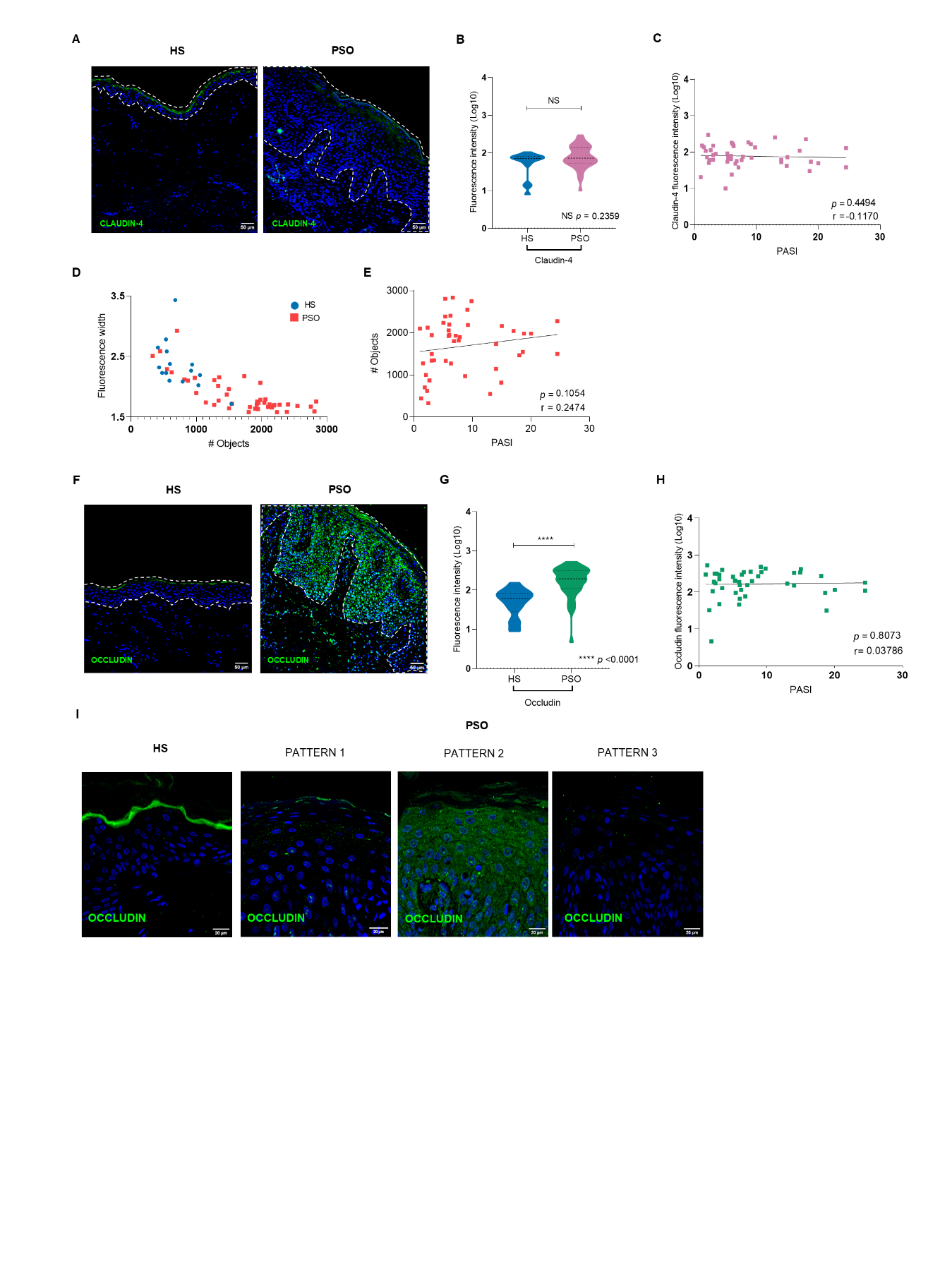


***Supplementary figure 1. Claudin-4 and occludin expression and distribution are not related to disease severity.***

A) Immunofluorescences of claudin-4 in healthy and psoriasis skin. B) Fluorescence intensity of claudin-4 and C) its correlation with PASI. D) Scatterplot of claudin-4 fluorescence width vs. # objects. E). Correlation of # objects with PASI. F) Fluorescence intensity of occludin in healthy and psoriasis skin. G) Fluorescence intensity of occludin and H) its correlation with PASI score. I) Immunofluorescences representing occludin distribution patterns in psoriasis patients.


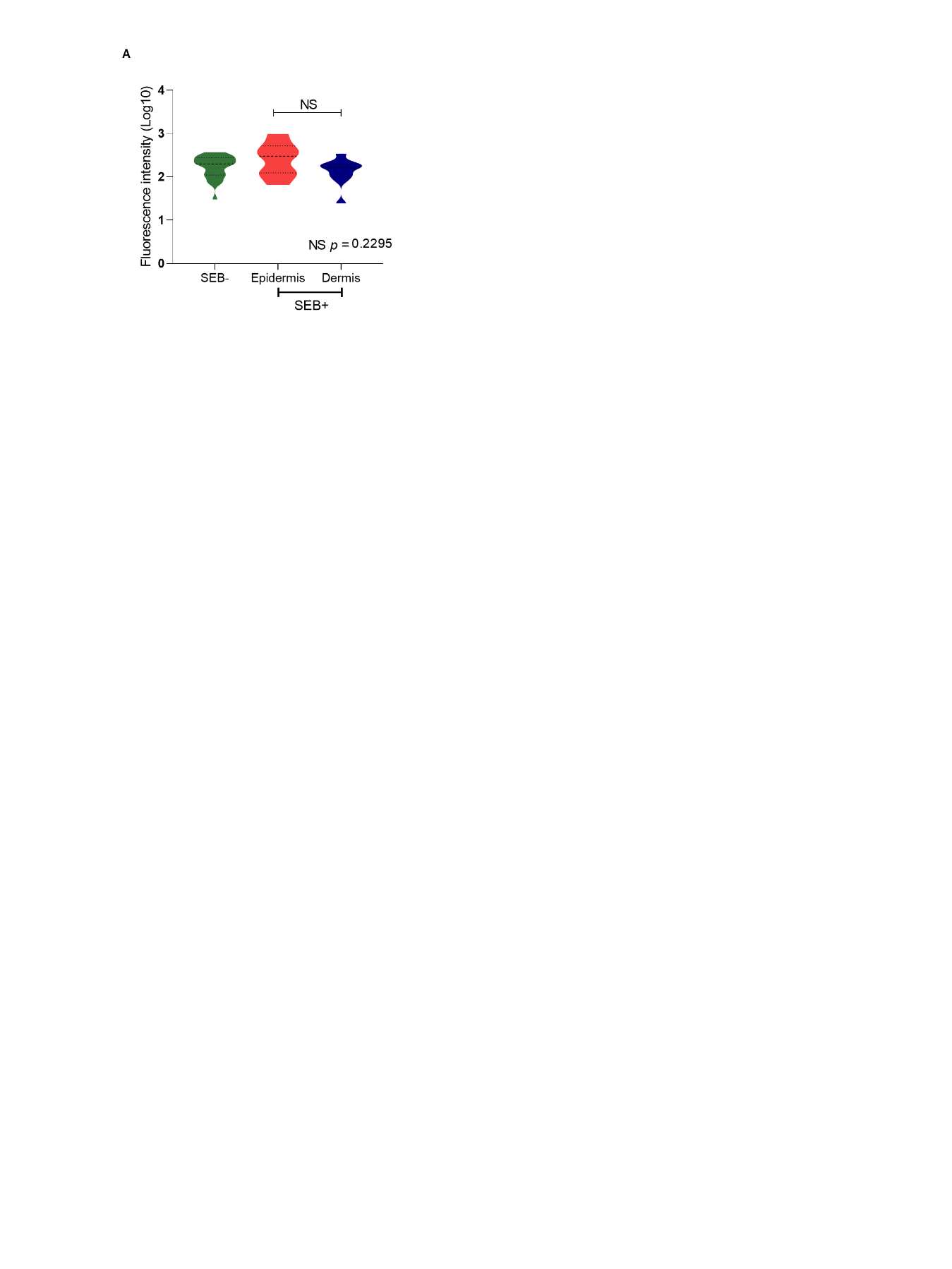


***Supplementary figure 2. SEB location in psoriatic skin is not related to claudin-1 fluorescence intensity***

A) Claudin-1 fluorescence intensity in SEB^-^ (n=27), SEB^+^ epidermis (n=7), and SEB^+^ dermis patients (n=10).


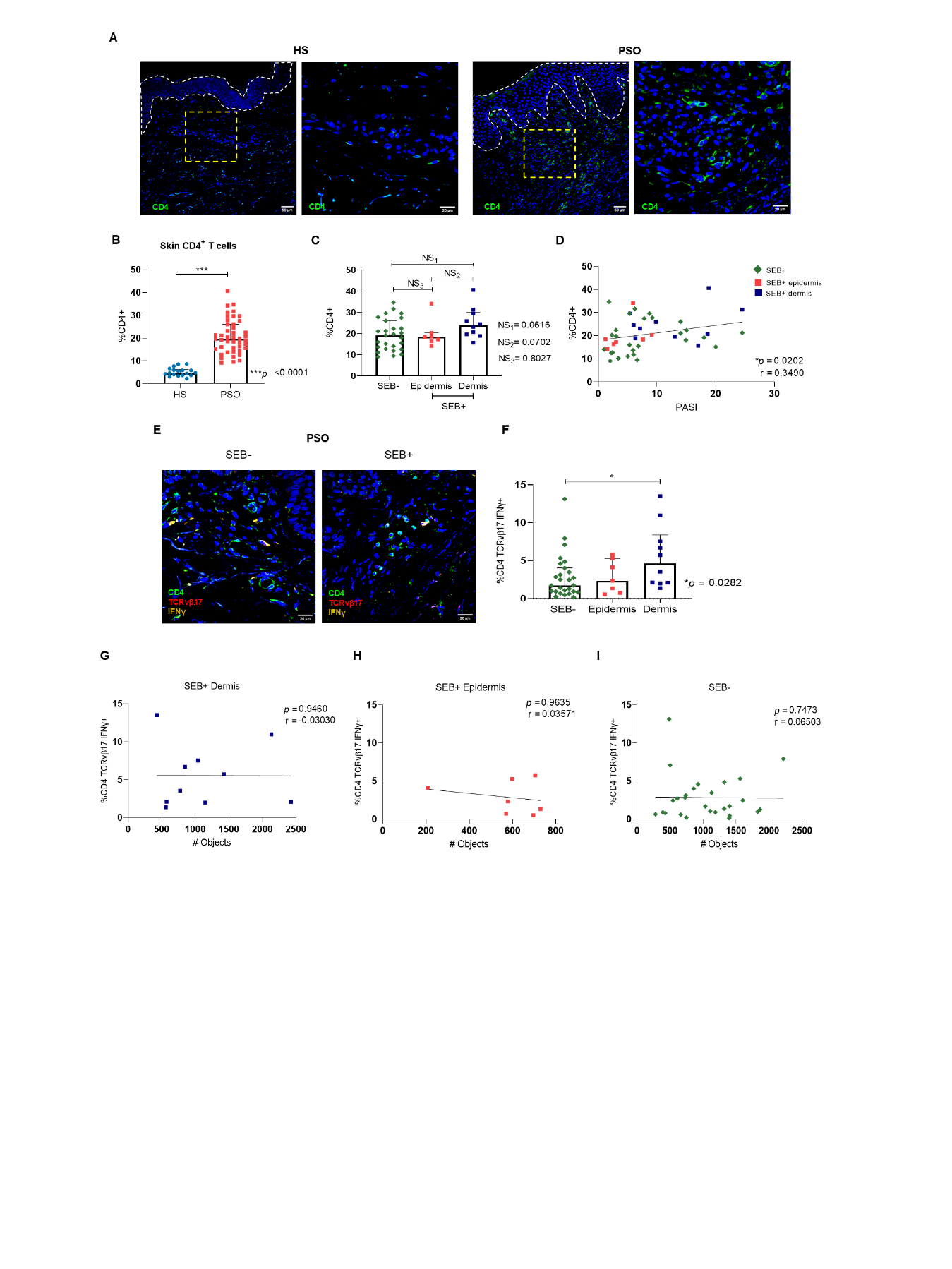


***Supplementary figure 3. CD4 T cells and CD4 TCRvβ17 IFN-γ cells do not distinguish SEB+ patients from SEB- patients.***

A) Immunofluorescences of CD4 T cells in healthy and psoriasis skin and B) its quantification. C) Percentage of CD4 T cells on SEB^-^, SEB^+^ epidermis, and SEB^+^ dermis patients and D) its correlation with PASI (n=44). E) Immunofluorescences of CD4 TCRvβ17 IFN-γ cells of SEB^-^ and SEB^+^ patients and F) its percentages (n=44). Correlation of CD4 TCRvβ17 IFN-γ cells with claudin-1 number of objects in G) SEB^+^ dermis (n=10), H) SEB^+^ epidermis (n=7) and I) SEB^-^ (n=27) patients.


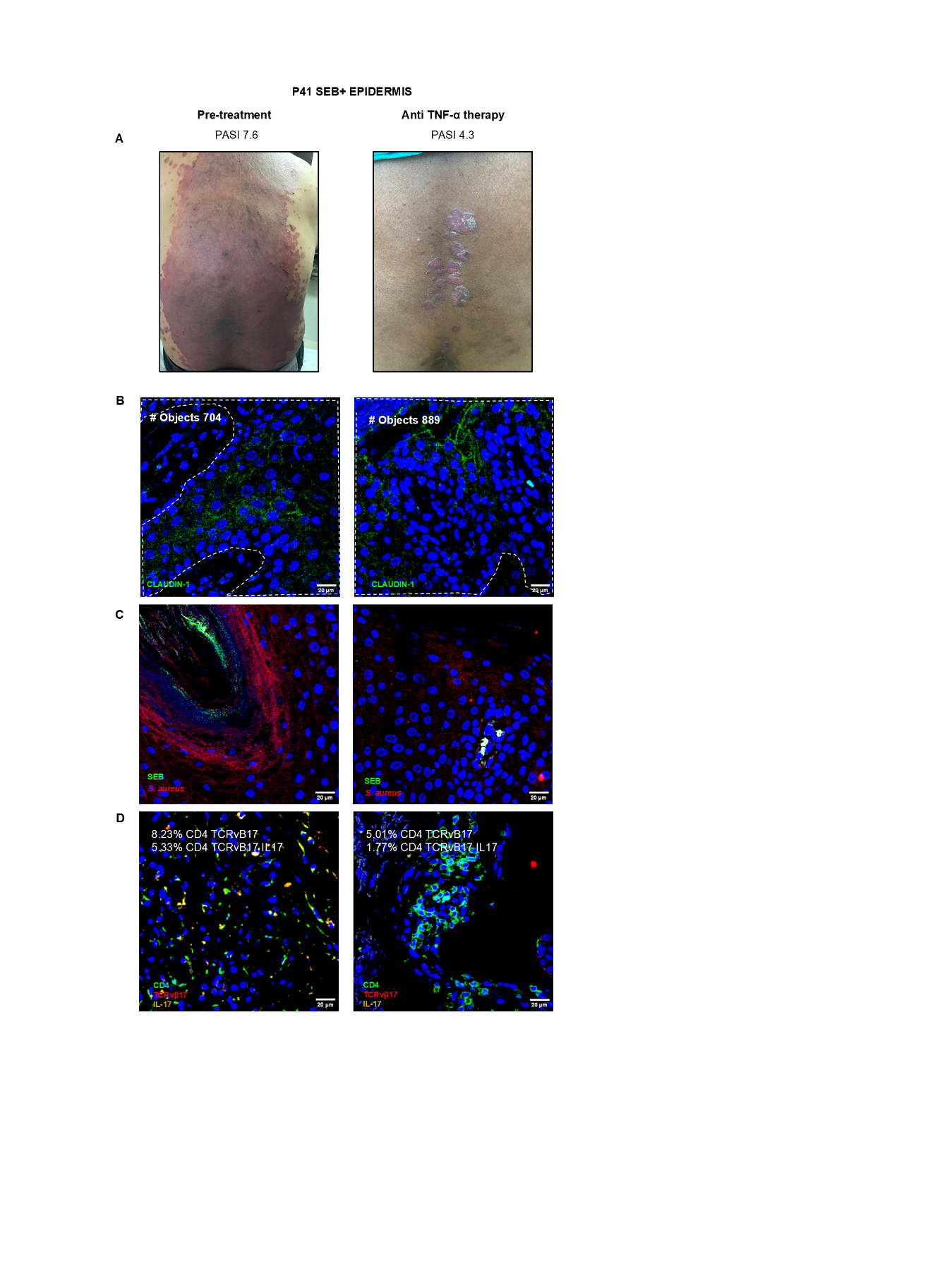


***Supplementary figure 4. Biological therapy modifies claudin-1 distribution, S. aureus, SEB, and CD4 TCRvβ17 infiltrate in psoriatic skin.***

A) Photography of the most representative skin lesion of SEB^+^ epidermis psoriasis patient pre-treatment (PASI 7.6) and after anti-TNFα Adalimumab therapy (PASI 4.3). B) Immunofluorescence images of claudin-1 distribution with the number of object values pre-treatment and after anti-TNFα therapy. C) Immunofluorescence images of *S. aureus* and SEB pre-treatment and after anti-TNFα therapy. D) Immunofluorescences images of CD4 TCRvβ17 IL-17+ cells with percentage values of CD4 TCRvβ17 cells and CD4 TCRvβ17 IL-17 cells, pre-treatment and after anti-TNFα therapy.
